# Lingual and Maxillary Labial Frenuloplasty with Myofunctional Therapy as a Treatment for Mouth Breathing and Snoring

**DOI:** 10.1155/2019/3408053

**Published:** 2019-03-10

**Authors:** Chirag Govardhan, Janine Murdock, Leyli Norouz-Knutsen, Sanda Valcu-Pinkerton, Soroush Zaghi

**Affiliations:** ^1^The Breathe Institute, Los Angeles, CA, USA; ^2^South County Pediatric Speech, Mission Viejo, CA, USA; ^3^UCLA Health, Santa Monica, CA, USA

## Abstract

Chronic mouth breathing may adversely affect craniofacial development in children and may result in anatomical changes that directly impact the stability and collapsibility of the upper airway during sleep. Mouth breathing is a multifactorial problem that can be attributed to structural, functional, and neurological etiologies, which are not all mutually exclusive. While therapeutic interventions (myofunctional, speech and swallowing, occupational, and craniosacral therapy) may address the functional and behavioral factors that contribute to mouth breathing, progress may sometimes be limited by restrictive lingual and labial frenum that interfere with tongue and lip mobility. This case report explores the case of a three-year-old girl with mouth breathing, snoring, noisy breathing, and oral phase dysphagia that was successfully treated with lingual and labial frenuloplasty as an adjunct to myofunctional therapy. Within four days of the procedure, the patient had stopped snoring and demonstrated complete resolution of open mouth breathing. The patient was also observed to have increased compliance with myofunctional therapy exercises. This report highlights the effectiveness of surgical interventions to improve the efficacy of myofunctional therapy in addressing open mouth posture and low tongue resting position.

## 1. Introduction

Open mouth breathing is a highly prevalent phenomenon that affects 10–25% of the pediatric population [1] with one study reporting a prevalence as high as 55% [2]. Mouth breathing for more than 10% of the total sleep time is considered pathologic [3, 4]. Patients who mouth breathe often exhibit signs of daytime sleepiness, lower rates of brain oxygenation, and immature auditory processing, which can increase their likelihood of having a learning disability [5–10]. Studies have shown that mouth breathing can adversely affect craniofacial growth patterns and can restrict the growth of the hard palate, leading to problems including airway instability and airway collapse [1, 3, 11]. Patients with mouth breathing have been observed to have lower academic achievement rates and poorer phonological working memory than controls [5, 12]. Mouth breathing has also been associated with a short lingual frenulum, which has been linked to obstructive sleep apnea (OSA) [13]. Pediatric patients with sleep-disordered breathing (SDB) or OSA can experience adverse effects on their behavior, neurocognition, memory, and rates of learning [14], highlighting the importance of addressing this problem at an early stage.

Abreu et al. describe three classifications of open mouth breathing, namely, *organic* (structural airway obstructions), *purely functional* (behavioral), and *special needs* (neurological factors) [2]. In practice, open mouth breathing often presents as a multifactorial problem with contributing factors from one or more classification domains. Enlarged tonsils and adenoids, nasal allergies, and a deviated septum are among structural etiologies that have been recognized to cause mouth breathing [15]. In addition, functional etiologies such as a low tongue resting posture and lips-apart open mouth posture may also physically manifest as mouth breathing. While myofunctional therapy has been shown to be an effective tool for the treatment of functional etiologies of mouth breathing and sleep-disordered breathing [16] by addressing posture and tone of the orofacial complex, structural restrictions of the lingual and labial frenulum have been observed to interfere with the efficacy and progress of therapy. Previous case reports and studies have shown tongue-tie releases to improve infant breast-feeding latch, increase milk transfer, and reduce maternal breast-feeding pain [17–19]. However, this is the first case report showing the role of lingual and labial frenuloplasty with myofunctional therapy in helping to improve symptoms of mouth breathing and noisy breathing.

## 2. Case Presentation

**Figure 1:**
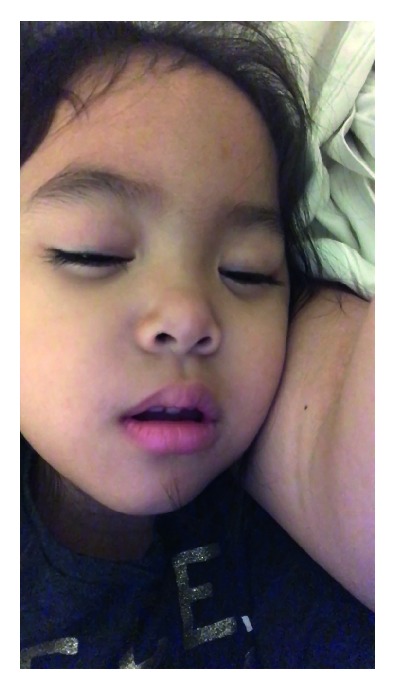
Patient sleeping with open mouth posture, noisy breathing, and incomplete lip seal (see complete video at https://tinyurl.com/Figure1Video).

The patient was a 3-year 7-month-old female referred by her speech-language pathologist, presenting with oromyofascial dysfunction characterized by speech sound production errors, difficulty swallowing, open mouth breathing, and noisy breathing during sleep. With respect to sleep, there were reports of difficulties going to sleep, waking up two to three times per night to drink water, getting up to go to the bathroom, open mouth breathing while asleep (Figure 1), snoring during sleep, and sweating more than usual during sleep. She experienced wheezing that was associated with asthma, which was treated with an Albuterol sulfate inhaler. There were reports of difficulty with effective chewing. In addition, the patient would eat around 50% of her meals, before refusing the rest. She experienced chronic cough and recurrent upper respiratory tract infections.

**Figure 2:**
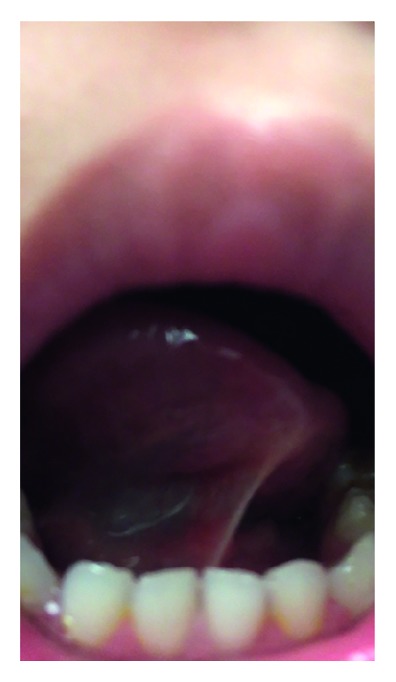
Lingual frenulum at time of physical exam, preoperative.

**Figure 3:**
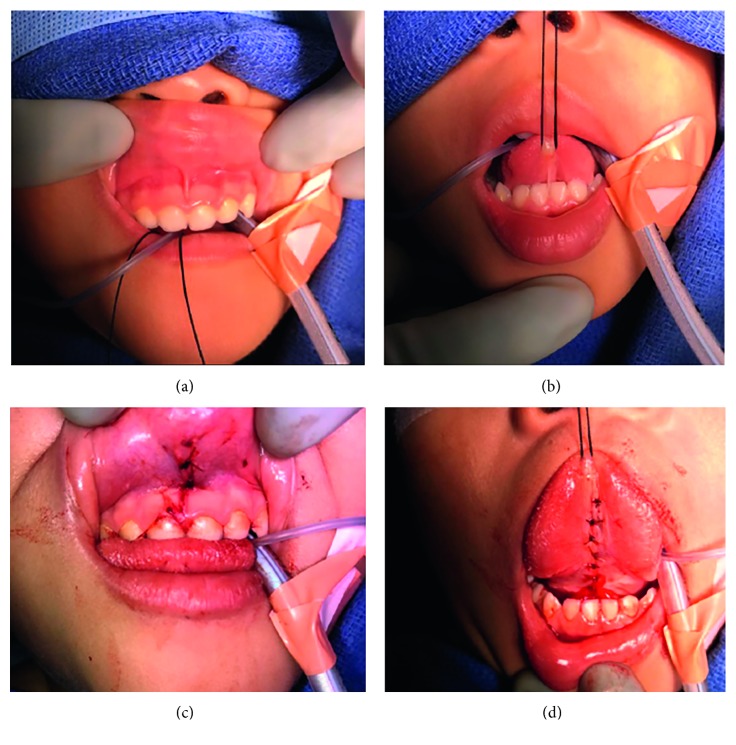
(a) Labial frenulum, preoperative. (b) Lingual frenulum, preoperative. (c) Labial frenulum, postoperative. (d) Lingual frenulum, postoperative.

Physical examination (Figure 2) of the patient showed her to be well developed, well nourished, and to appear the stated age. The patient was alert, oriented, able to communicate, and respond appropriately to questions. During the nasal examination, the nose had no external deformity. The nasal septum was straight, and the inferior turbinates were grade 2 bilaterally. There were class 3 dental occlusion and a class 3 facial-skeletal relationship characteristic of anterior-posterior maxillary deficiency. Oropharyngeal examination showed grade 3 modified Mallampati tongue position and grade 2 tonsils. The patient was found to have a restrictive class 2 upper labial frenulum with tethering of the upper lip (Figure 3(a)) and restrictive grade 4 lingual frenulum [20] (Figure 3(b)).

Based on the patient history and the physical examination, the assessment revealed that the patient had functional and structural mouth breathing due to open mouth posture and low tongue posture in the setting of restrictive labial and lingual frenulum. The risks and benefits of lingual and labial frenuloplasty to facilitate lip closure and proper tongue resting posture were discussed with the parents and included, but were not limited to pain, inflammation, bleeding, scarring, need for revision surgery, and failure for significant improvement. An informed consent document was signed by the parents.

The maxillary labial frenuloplasty was performed under general anesthesia. Local anesthesia was achieved by applying 1 cc of 0.25% Marcaine with 1 : 200000 epinephrine to the maxillary labial frenulum via a 27-gauge needle. Pressure was applied lateral to the frenulum to locate the point of maximum tension. The maxillary labial frenulum was incised at the base of attachment with the use of sharp scissors. A V-to-Y lip closure was performed with a 4-0 chromic suture applied in a simple interrupted fashion (Figure 3(c)).

The lingual frenuloplasty procedure was then performed. A 2-0 silk suture was applied to the tip of the tongue as a retraction stitch. Local anesthesia was achieved by applying 1 cc of 0.25% Marcaine with 1 : 200000 epinephrine to the lingual frenulum via a 27-gauge needle. The tongue was retracted in the anteroposterior direction extending to the roof of the mouth and maxillary central incisors. Tension was applied to the floor of the mouth to protect the floor of mouth salivary glands, as well as Wharton's duct. A hemostat was used to clamp the restrictive lingual frenulum 5 mm above the attachments of the sublingual gland duct. The fibrous band was gently excised with the use of iris scissors. The underlying myofascial fibers of the genioglossus muscle were dissected further, with blunt cotton tips and sharp iris scissors being used to release the muscle from the overlying mucosa. The dissection was continued until there was adequate improvement to the tongue range of motion such that the tongue could extend up towards the maxillary central incisors in maximal mouth opening position. Simple interrupted sutures were used to close the diamond-shaped defect into a vertical line, as a means to lengthen the ventral tongue, with 4-0 chromic suture applied in a simple interrupted fashion. In total, the tongue was released from a grade 4 restricted range of motion to a grade 1 range of motion (Figure 3(d)). All wounds were hemostatic at the completion of the procedure. The patient was gently awoken from anesthesia and taken to recovery in stable condition.

**Figure 4:**
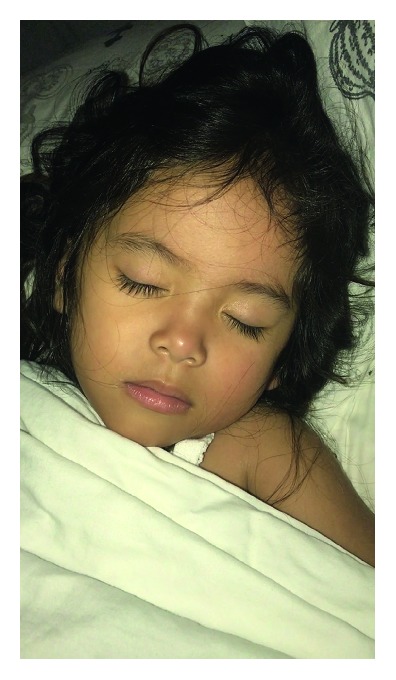
Patient sleeping with closed mouth posture and exclusive nasal breathing (see complete video at https://tinyurl.com/Figure4Video).

The patient returned to the clinic four days after the procedure. The wound sites were healing appropriately, and there were no postoperative complications observed. The patient's mother reported that within the first day of returning home, the patient's issues with chewing had improved significantly, and she was more interested in eating different foods. In addition, her appetite appeared to have increased, and the patient would complete her entire meal before asking for more food (as compared to having only eaten around 50% before treatment). By the fourth day after surgery, the patient exhibited closed-mouth nasal breathing while asleep (Figure 4). There were no longer any observed events of snoring and/or noisy breathing. The mother reported that the patient had remained compliant with myofunctional and speech therapy.

**Figure 5:**
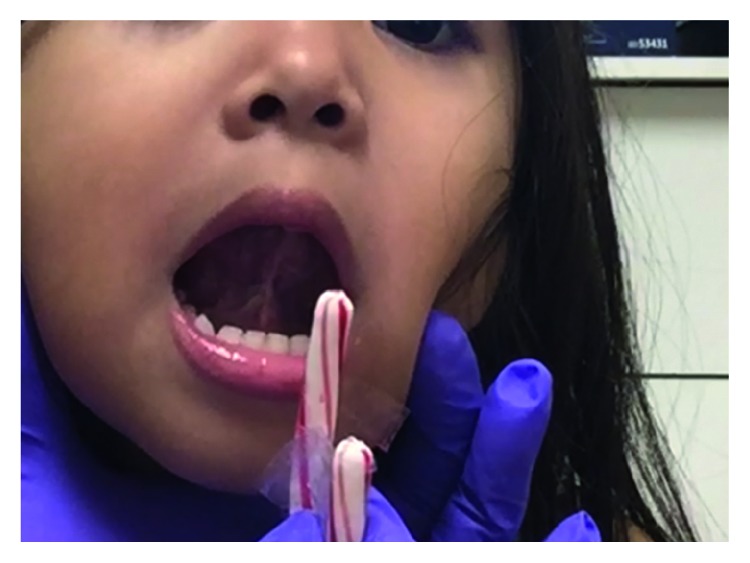
Lingual frenulum, 2 months postop.

The patient returned for a 2-month postoperative visit. During the examination at this visit, no scar tissue was observed, and the wound sites had closed. Grade 1 tongue range of motion was observed (Figure 5).

The patient's family wrote in a letter to the clinic approximately six months after the procedure, mentioning that the patient was doing very well with no complications. In addition, the patient had been reported to have completely stopped mouth breathing and snoring while asleep. However, the patient was reported to still have occasional episodes of cold and cough, as well as one episode of asthma exacerbation. Finally, the patient was reported by her family to have made progress with myofunctional and speech therapy, but the goals of eliminating the tongue thrust, achieving proper resting posture, and improving speech sound production errors were not met due to early discontinuation of treatment.

## 3. Discussion

In this report, we have detailed the case of a 3-year 7-month-old female who presented with mouth breathing and noisy breathing during sleep despite a completely patent nasal cavity, who was successfully treated with labial and lingual frenuloplasty accompanied with myofunctional therapy. Myofunctional therapy aims at addressing functional issues that can contribute to and exacerbate mouth breathing through therapeutic exercises, self-awareness, and supportive techniques to improve tongue posture, lip seal, and nasal patency [16, 21, 22].

Once nasal patency is achieved or is not an issue, such as in this case, progress with myofunctional therapy to improve mouth breathing may be limited due to restrictions in the lingual and labial frenulum. This case highlights the role of surgical interventions to help improve oral and tongue posture among patients who seek myofunctional therapy as a treatment for mouth breathing issues. However, it should be noted that pre- and postoperative myofunctional therapy is essential for optimal wound healing and long-term reeducation of orofacial functions, including chewing, swallowing, oral rest posture, and nasal breathing. While the risks are not fully identified, clinically, issues such as a tongue thrust, open mouth posture, and speech production errors may still persist after frenuloplasty and may respond to myofunctional therapy.
